# West London Maternity Trauma and Loss Care Service 2022/2023 Evaluation

**DOI:** 10.1192/bjo.2024.508

**Published:** 2024-08-01

**Authors:** Jasmine Reed, Sanne Van Rhijn, Hadiss Khossravi

**Affiliations:** West London NHS Trust, London, United Kingdom

## Abstract

**Aims:**

The Maternity Trauma and Loss Care Service provides specialist care to women and birthing people who are affected by birth trauma, baby loss and severe fear of childbirth. The service has an integrated team of specialist midwives and psychological practitioners. This evaluation is the first, to our knowledge, to describe the challenges and successes of setting up a Maternal Mental Health Service as depicted in the NHS Long Term Plan.

**Methods:**

The sample includes all women and birthing people who were referred to the service over the 12-month period from 1^st^ April 2022 to 31^st^ March 2023.

A mixed-methods design was used to explore and interpret the delivery of the service. Descriptive data was used to describe basic service information: client demographics, time from referral to assessment and numbers accessing treatment. Quantitative data from pre and post clinical measures to look at symptom change over the treatment period. Qualitative data to capture the experience of clients.

**Results:**

The service received 254 referrals between April 2022 and March 2023. For primary referral reasons of accepted clients, 92 clients (50%) were referred due to perinatal trauma, 65 clients (35%) were referred for perinatal loss, 26 clients (14%) for Tokophobia and 2 were referred for other reasons (1%). Three quarters of referrals were accepted and 99 (53%) were pregnant at the time of referral. 53 clients (29%) were postnatal, 32 clients (17%) were post-loss and one was pre-conception.

Of the interventions offered, 49% were offered a midwifery intervention, 31% a psychology intervention and 18% were offered midwifery and psychology treatment. A small number attended groups. 36 clients referred during this 12-month period completed treatment.

PTSD Checklist for DSM-5 scores and Clinical Outcomes in Routine Evaluation scores indicate that service users experienced a reduction of symptoms between the start and end of treatment. There was an average reduction in scores on the PTSD-checklist of 17 and on the Clinical Outcomes in Routine Evaluation of 6.4. 17 clients completed the service satisfaction survey, all of which were positive about the service and its impact on wellbeing.

**Conclusion:**

The Maternity Trauma and Loss Care service continues to fill the gap identified in the long-term plan providing a much needed integrated service to women and birthing people who experience trauma and loss on their reproductive journeys. Areas identified for service development include further developing a pathway for peer support and partners.

